# *QuickStats*: Percentage* of Children^†^ Aged 2–17 Years With >2 Hours of Screen Time Per Weekday,^§^ by Sex and Age Group — National Health Interview Survey,^¶^ United States, 2020

**DOI:** 10.15585/mmwr.mm7103a6

**Published:** 2022-01-21

**Figure:**
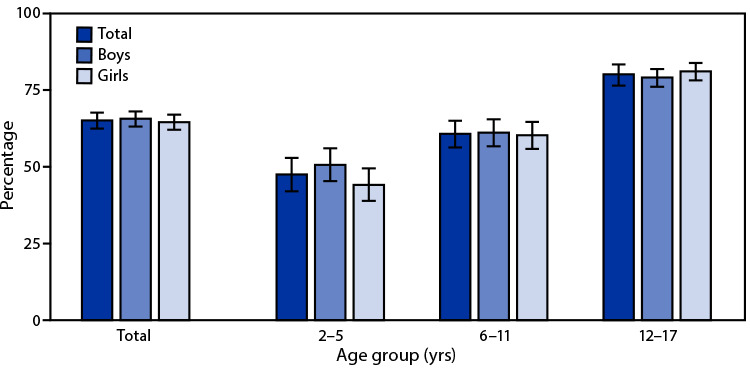
Overall, 65.7% of boys and 64.6% of girls aged 2–17 years spent >2 hours of screen time per weekday, in addition to screen time spent for schoolwork. Among both boys and girls, the percentage of children who spent >2 hours of screen time increased with increasing age group from 47.5% for those aged 2–5 years to 80.2% for those aged 12–17 years.

**Source:** National Center for Health Statistics, National Health Interview Survey, 2020. https://www.cdc.gov/nchs/nhis/index.htm

